# Apoptotic gene loss in Cnidaria is associated with transition to parasitism

**DOI:** 10.1038/s41598-023-34248-y

**Published:** 2023-05-17

**Authors:** Alexander M. Neverov, Alexander Y. Panchin, Kirill V. Mikhailov, Marina D. Batueva, Vladimir V. Aleoshin, Yuri V. Panchin

**Affiliations:** 1 Department of Bioengineering and Bioinformatics, Lomonosov Moscow State University, Moscow, Russian Federation 119234; 2 Kharkevich Institute for Information Transmission Problems, Russian Academy of Sciences, Moscow, Russian Federation 127994; 3 Belozersky Institute of Physico-Chemical Biology, Lomonosov Moscow State University, Leninskiye Gory 1-40, Moscow, Russian Federation 119991; 4 Institute of General and Experimental Biology Siberian Branch of Russian Academy of Sciences, Ulan-Ude, Russian Federation 670047

**Keywords:** Cancer, Cell biology, Computational biology and bioinformatics, Evolution, Molecular biology

## Abstract

The phylum Cnidaria consists of several morphologically diverse classes including Anthozoa, Cubozoa, Hydrozoa, Polypodiozoa, Scyphozoa, Staurozoa, and Myxozoa. Myxozoa comprises two subclasses of obligate parasites—Myxosporea and Malacosporea, which demonstrate various degrees of simplification. Myxosporea were previously reported to lack the majority of core protein domains of apoptotic proteins including caspases, Bcl-2, and APAF-1 homologs. Other sequenced Cnidaria, including the parasite *Polypodium hydriforme* from Polypodiozoa do not share this genetic feature. Whether this loss of core apoptotic proteins is unique to Myxosporea or also present in its sister subclass Malacosporea was not previously investigated. We show that the presence of core apoptotic proteins gradually diminishes from free-living Cnidaria to *Polypodium* to Malacosporea to Myxosporea. This observation does not favor the hypothesis of catastrophic simplification of Myxosporea at the genetic level, but rather supports a stepwise adaptation to parasitism that likely started from early parasitic ancestors that gave rise to Myxozoa.

## Introduction

Multicellular organisms can get rid of old, damaged, unuseful or precancerous cells by apoptosis, a form of programmed cell death or cell suicide. Key apoptotic proteins include proteolytic enzymes called caspases, which trigger cell death by cleaving specific cellular proteins^1^. Inactive precursors, or procaspases, are activated through cleavage by other caspases^2^. Extracellular or intracellular death signals can initiate this proteolytic cascade under tight regulation of adaptor and regulatory proteins such as Fas-associated death domain protein (FADD) and Bcl-2 family proteins^3,4^.

Variation exists between the apoptotic pathways of invertebrates. For example, the Pacific oyster has mammalian-like apoptotic pathways^5^, whereas, *Caenorhabditis elegans* does not use cytochrome C for the apoptotic caspase cascade activation^6^. Apoptosis has been described and well-studied in model free-living Cnidaria, such as *Hydra*^7^ which has numerous caspases, Bcl-2 protein family members, an APAF-1 homolog, components of a putative death receptor pathway and inhibitors of apoptotic proteases. Like other animals, *Hydra* is capable of developing tumors^8^ and the transcriptomes of these tumors reveal misregulation of genes related to mammalian apoptosis genes.

Let us review the general features of the better-understood mammalian apoptosis, keeping in mind that similar actors can be found in free-living Cnidaria^7,9^. There are two principal apoptotic pathways: the extrinsic pathway and intrinsic pathway^1^. In its simplified version, the extrinsic apoptotic pathway starts with external signals that activate Death domain harboring transmembrane receptors (TNFR/Fas)^1^. This signal is transduced via adaptor proteins such as TRADD/FADD with Death and Death-effector domains that transform initiator Death-effector domain containing procaspases into active initiator caspases which induce the apoptosis caspase cascade^1^.

The intrinsic apoptotic pathway is activated in response to various oncogenes and intracellular stressors, including DNA damage, reactive oxygen species, endoplasmic reticulum stress, and hypoxia^1^. The activation is mediated via transcription factors such as p53^10^. In response BH3-only Bcl-2 family proteins are expressed, leading to the inhibition of anti-apoptotic Bcl-2. This allows liberated Bak proteins to form channels within the mitochondrial membranes causing cytochrome C release. Apoptotic protease activating factor 1 (APAF-1) proteins bind cytochrome C with their WD40 repeats and then bind with each other via their CARD domains and form the apoptosome^1^. The apoptosome activates the CARD-domain containing initiator procaspases of the intrinsic apoptotic pathway causing the apoptotic caspase cascade^11^. Inhibitors of apoptosis proteins (IAPs) inhibit caspases^12^. More detailed descriptions of apoptosis are reviewed elsewhere^1^.

**Figure 1:**
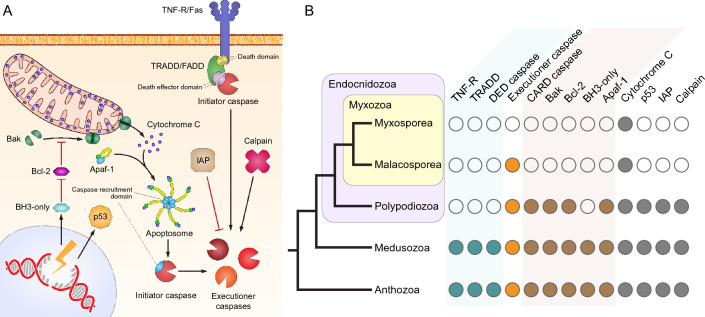
(**A**) Scheme of extrinsic and intrinsic apoptotic pathways showing the main actors considered in this article. (**B**) Consensus cladogram of cnidarians relationships and presence of main actors of apoptosis in studied free-living Cnidaria, *Polypodium*, Malacosporea, and Myxosporea. Missing actors are shown with empty circles. Note that among Malacosporea caspases were only found in *Buddenbrockia*. Compared to free-living Cnidaria, *Polypodium* lacks core proteins involved in the extrinsic apoptosis pathway. For the intrinsic pathway *Polypodium* has 1 predicted intrinsic pathway initiator caspase and 1 executioner caspase, compared to 2 and 11 in *Hydra* respectively. Myxosporea universally lack all main actors of apoptosis except cytochrome C, whose gene is however absent in *Myxobolus squamalis*, *Henneguya salminicola* and is a pseudogene with multiple inner stop-codons in *Kudoa iwatai*, *Sphaeromyxa zaharoni*, and *Enteromyxum leei*. Note that *Henneguya salminicola* apparently lacks a mitochondrial genome and the respiratory chain^24^.

Surprisingly, Myxosporea (see Fig. 1B for simplified cladogram) species *Thelohanellus kitauei*, *Kudoa iwatai*, *Myxobolus pronini*, *Sphaeromyxa zaharoni*, *Enteromyxum leei* were reported to completely lack a number of Pfam-domains belonging to key apoptotic proteins including caspases, Bcl-2, APAF-1, and p53 homologs^13^. This finding was interpreted in favor of the SCANDALs (Speciated by CANcer Development AnimaLS) hypothesis that suggested that Myxosporea evolved from a transmissible cancer and have undergone catastrophic simplification. The limitations of that study were the consideration of Pfam-domains only, but not complete genes and that genetic data for the sister subclass Malacosporea was unavailable.

Unlike Myxosporea, Malacosporea are unlikely candidates for the SCANDAL hypothesis because of their more sophisticated morphology and development stages similar to gastrulation^14^. Malacosporean species, *Tetracapsuloides bryosalmonae*, can form ~ 350 µm spherical spore sacs^15^ while another malacosporean, *Buddenbrockia plumatellae*, forms 1 mm long worm-like sacs with muscle cells that are capable of active movement in their bryozoan hosts^16^. In the fish host these species form a pseudoplasmodial structure in renal tubules^15,17^. *Tetracapsuloides bryosalmonae* causes a proliferative kidney disease in salmonids which affects important wild and aquacultural populations^18^. Two other described, but less studied species of Malacosporea demonstrate similar morphology and structure^14,19^.

Recently transcriptomic data for members of Malacosporea *Buddenbrockia plumatellae* and *Tetracapsuloides bryosalmonae* became available^20–23^. Also, genomic data became available for additional Myxosporea species: *Henneguya salminicola*, *Myxobolus squamalis*^24^, and *Myxobolus honghuensis*^25^.

Notably, the *Henneguya salminicola* has been shown to completely lack a mitochondrial genome which is especially interesting in the context of the apparent loss of apoptosis in Myxosporea species^24^. We decided to complete this data with our own sequences of *Polypodium hydriforme* and *Myxobolus pronini* to comprehensively study the loss of apoptosis-related genes in parasitic Cnidaria.

In this article we applied a number of comparative genomic methods, such as BLAST^26^ for homology search, functional domain prediction using HMMER^27^, phylogenetic analysis, and filtering techniques to available genomic and transcriptomic data of Myxosporea, Malacosporea, Polypodiozoa, and free-living Cnidaria to establish the evolutionary gain and loss of their apoptosis-related genes. We show that the SCANDAL hypothesis for Myxosporea origin is weakened by the observation that Malacosporea also lost many genes involved in apoptosis regulation, although to a lesser extent. Our analysis reveals and describes in detail the gradual process of apoptosis-related gene loss in Myxozoa that appears to have started in the common ancestor of both Myxosporea and Malacosporea subclasses.

## Results

### Presence of genes coding key apoptotic proteins in parasitic Cnidaria

Comparison of cnidarian species reveals that the transition to parasitism is associated with gradual loss of key apoptotic factors in the parasitic classes Polypodiozoa and Malacosporea, up to a complete reduction of almost all pathway components in Myxosporea (Fig. 1 and Table 1). We chose a ‘standard’ mammalian-like apoptotic pathway as a reference, because comparative genomic studies find many similarities between it and the predicted apoptotic pathway of free-living Cnidaria such as *Hydra*^9^.

**Table 1:** Presence of main actors of apoptosis in Cnidaria and other animals.

	Myxosporea	Malacosporea	*Polypodium hydriforme*	Free-living Cnidaria (*Hydra vulgaris*)	*Caenorhabditis elegans*	*Homo sapiens*
Caspases	–	BuddCasp	PolCasp, PolCARDCasp	HyCaspA, B, C, D, E, F, G, H, I, L, M HyCARD 1, 2 HyDEDCasp HyDDCasp	Ced-3, Csp-1, Csp-2	Caspase-1, 2, 3, 4, 5, 6, 7, 8, 9, 10, 12, 13, 14
Bcl-2 multidomain	–	–	PolBCL-2–1, 2, PolBOK, PolBAK-1, 2	HyBcl-2-like 1, 2, 3, 4, 5, 6, 7, HyBak-like 1, 2	Ced-9	Bcl-2, Bcl-xL, Bcl-W, Mcl-1, Bcl-B, Bcl-2A1, Bax, Bak, Bok
BH3-only	–	–	–	HyBH3-only 1, 2, 3, 4	Egl-1, Ced-13	Bid, Bmf, NOXA, PUMA, Bad, Bim, Bik, Hrk
Death receptor	–	–	–	HyTNFR-like receptor	–	TNF-R and others
Adaptor protein	–	–	–	HyFADD	–	FADD, TRADD
IAP	–	–	PolIAP	HyIAP	–	XIAP, NAIP, c-IAP1, 2
APAF-1	–	–	PolAPAF-1	HyAPAF-1	Ced-4	APAF-1
p53	–	–	PolP53	p53	Cep-1	p53
Cytochrome C	MpCytC, TkCytC, MhCytC, ElCytC*, SzCytC*, KiCytC* Absent in: *H. salminicola M. squamalis*	BuddCytC, TetrCytC	PolCytC	Cytochrome C	CPS-6, WAH-1	Cytochrome C
Calpain	–	–	PolCalp-5, 7, PolClassCalp	Calpain-5, 7, 9, and others	CLP-1, TRA-3	Calpain-5, 7, 9, and others

Data for *H. vulgaris*, *C. elegans*, and *H. sapiens* was taken from^1,6,7,28–31^.

The table contains information of the species from which a protein was found (Mp = *Myxobolus pronini*, El = *Enteromyxum leei*, Tk = *Thelohanellus kitauei*, Sz = *Sphaeromyxa zaharoni*, Ki = *Kudoa iwatai*, Mh = *Myxobolus honghuensis*, Budd = *Buddenbrockia plumatellae*, Tetr = *Tetracapsuloides bryosalmonae*, Pol = *Polypodium hydriforme*). *—these genes contain stop-codons. Note that *C. elegans* has an apoptotic pathway that involves homologs of mammalian BH3-only, BCL-2-like^32^, Apaf-1, and caspases, but that pathway does not involve cytochrome C release^6^.

The free-living Cnidaria *Hydra vulgaris* was previously known to share all key components of both the intrinsic and extrinsic apoptotic pathways (Table 1). We found that the parasitic Cnidaria *Polypodium hydriforme* lost the main components of the extrinsic apoptotic pathway: it completely lacks the Death domain required for the extrinsic receptor and adaptor proteins as well as the Death-effector domain required for the extrinsic initiator caspase. However, it maintains most of the intrinsic apoptotic pathway components with the exception of BH3-only Bcl-2 family members. Also, we were able to detect only one predicted intrinsic pathway initiator (CARD domain containing) caspase and one executioner caspase compared to two and eleven found in *Hydra*. Malacosporea have undergone further loss of apoptosis-related proteins including p53, all Bcl-2 family members, APAF-1, CARD domain, and DEATH domain containing proteins (see Supplementary tables, Supplementary table S2, for full list of domains). One caspase without a CARD domain was found in *Buddenbrockia*. Myxosporea universally lack all main actors of apoptosis except cytochrome C, whose gene is however absent in *Myxobolus squamalis* and *Henneguya salminicola*, and is a pseudogene with multiple inner stop-codons in *Kudoa iwatai*, *Sphaeromyxa zaharoni*, and *Enteromyxum leei*. *Henneguya salminicola* apparently entirely lacks a mitochondrial genome^24^. Detailed findings and a comparison with other animals are presented in Table 1.

This pattern of apoptotic-protein loss among parasitic Cnidaria is consistent with gradual loss-of-function in contrast to previous findings suggesting catastrophic loss of apoptotic and cancer-related proteins in Myxosporea^13^. While this study focuses only on cnidarian apoptosis, we observe a gradual decline in the number of cancer-related PFAM domains in order from free-living Cnidaria to *Polypodium* to Malacosporea to Myxosporea (see Supplementary tables, Supplementary table S1).

### Number of caspases gradually diminishes in parasitic Cnidaria

**Figure 2:**
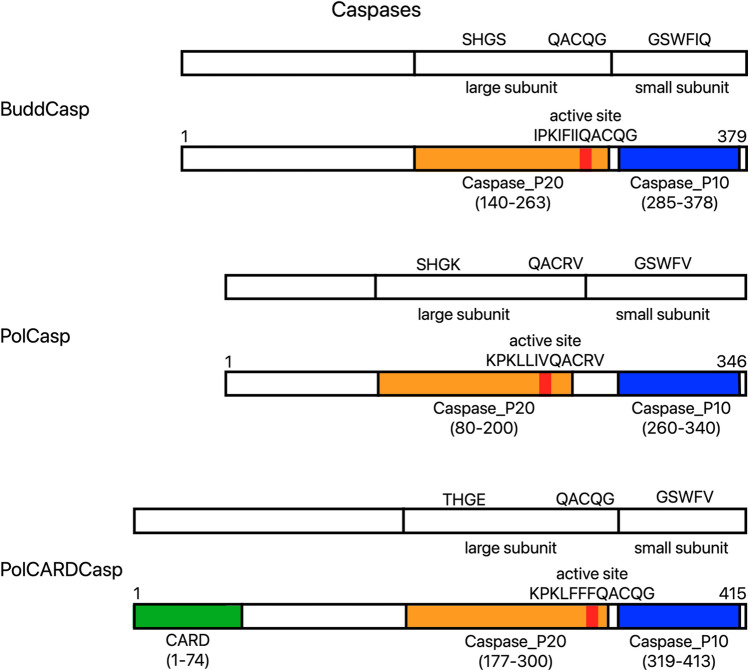
Predicted domain structures of caspases identified in *Polypodium* and *Buddenbrockia*. All three predicted proteins contain conserved amino acid sequences typical for caspases. One of the caspases in *Polypodium* contains a CARD domain indicating its possible role as an initiator in the intrinsic pathway. The transcriptome of *Buddenbrockia plumatellae* (Malacosporea) contains one potentially functional sequence encoding a caspase homolog, while *Tetracapsuloides bryosalmonae* (also Malacosporea) and all investigated species of Myxosporea contain none. (Pol—*Polypodium hydriforme*, Budd—*Buddenbrockia plumatellae*, Casp—caspase, CARD—caspase recruitment domain, Caspase_P20—caspase family p20 domain, Caspase_P10—caspase family p10 domain).

Caspases are proteases that play a crucial role in both intrinsic and extrinsic apoptotic pathways. Our results suggest the complete absence of caspases not only in Myxosporea, but also in one of two analyzed members of Malacosporea (*Tetracapsuloides bryosalmonae*). The other Malacosporea used in our analysis (*Buddenbrockia plumatellae*) has only one caspase (Fig. 2) in contrast to the free-living *Hydra* that has fifteen^7^. Based on domain composition the single *Buddenbrockia* caspase is an executioner caspase and not an initiator (CARD-domain containing) caspase, thus insufficient to trigger either of the apoptotic pathways. In contrast *Polypodium* has both an initiator and an executioner caspase, allowing for the classical intrinsic apoptotic pathway to work. Based on phylogenetic analysis (see Supplementary materials, Fig. S3 for phylogenetic tree) we can conclude that the CARD domain containing caspase from *Polypodium* is homologous to the CARD domain containing caspases of *Hydra* HyCARDCasp-1 and HyCARDCasp-2. And the only identified executioner caspase in *Polypodium* is unlikely to be orthologous to the one found in *Buddenbrockia*. The loss of extrinsic apoptotic pathways in Malacosporea, Myxosporea, and *Polypodium* can be predicted from the lack of DEATH receptors, adaptor proteins, and Death-effector domain containing initiator caspases.

### In contrast to *Polypodium*, Myxozoa likely lack Bcl-2 family members

**Figure 3:**
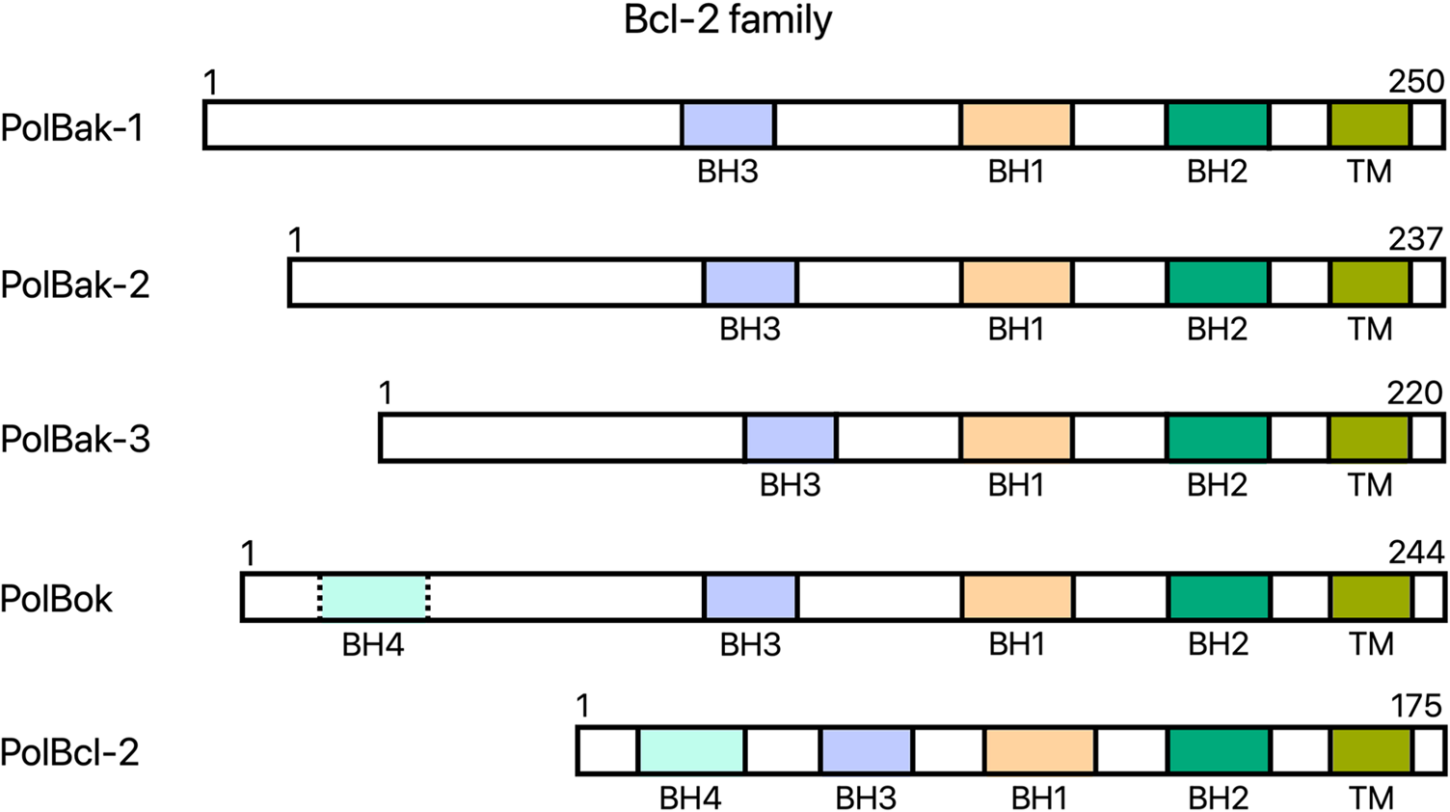
Predicted domain structures of Bcl-2 family proteins in *Polypodium*. Phylogenetic analysis suggests that two of these proteins are related to BAK pro-apoptotic proteins. The classification of three other Bcl-2 family proteins is more complicated, however we can say that one of them contains the BH4 domain typical for anti-apoptotic Bcl-2 and pro-apoptotic BOK proteins. The last two Bcl-2 family proteins lack the BH4 domains despite being classified as BOK and anti-apoptotic Bcl-2 proteins by InterProScan. (Pol—*Polypodium hydriforme*, BH1-4—Bcl-2 homology domains 1–4, TM—transmembrane domain).

The Bcl-2 family plays an important role in regulating the intrinsic apoptotic pathway. Some Bcl-2 family members are proapoptotic (Bak and Bok proteins in humans) and others are anti-apoptotic (human Bcl-2)^33^. Our analysis suggests that Myxozoa lack any Bcl-2 protein members, consistent with the predicted lack of intrinsic apoptotic pathways. In *Polypodium* we predicted five Bcl-2 protein family members and attempted to infer their pro- or anti-apoptotic roles based on similarity with known human and *Hydra* Bcl-2 family protein members (Fig. 3, see Supplementary materials, Fig. S1 for phylogenetic tree). One *Polypodium* Bcl-2 protein was predicted as a pro-apoptotic Bok homologue by InterProScan^34^. The separation between Bcl-2 and Bak proteins is based on phylogenetic analysis (see Supplementary materials, Fig. S1). Overall, the results favor the existence of an intrinsic apoptotic pathway in *Polypodium* and its lack in Myxozoa.

### Cytochrome C is likely lost in some myxosporeans

Predicted genes for cytochrome C were found in *Polypodium*, Malacosporea (both *Tetracapsuloides* and *Buddenbrockia*), and Myxosporea. However, in Myxosporea the situation with cytochrome C is rather complex. In *Myxobolus pronini*, *M. honghuensis*, and *Thelohanellus kitauei* the predicted cytochrome C proteins share high similarity with each other. Although, cytochrome C could not be found in a published *Myxobolus cerebralis* transcriptome (GBKL00000000.1). Also, we were unable to identify any potential genes of cytochrome C in the available genomes and transcriptomes of *Henneguya salminicola* and *Myxobolus squamalis*. Note that *Henneguya salminicola* was previously shown to lack a mitochondrial genome so this is a special case^24^.

**Figure 4:**
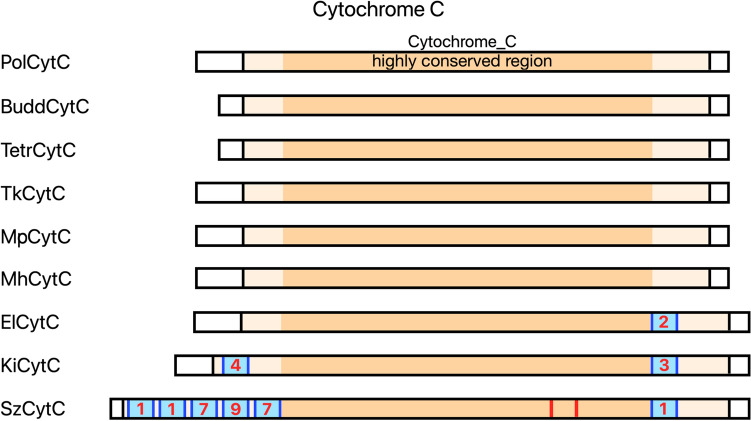
Cytochromes C genes in parasitic Cnidaria. The InterPro Cytochrome_C domain is shown with a light color. Within this domain a highly conserved region is colored with a darker color. Red lines show stop codons, blue regions mark insertions, and red numbers within these insertions represent the number of stop codons located in the respective insertion. Note that *Myxobolus honghuensis* has 5 predicted cytochrome C genes, 2 of them are identified in both the transcriptome and genome, while 3 others were found only in the genome (and have several stop codons in the predicted sequences of their structural domains similar to ElCytC). (CytC—cytochrome C, Pol—*Polypodium hydriforme*, Budd—*Buddenbrockia plumatellae*, Tetr—*Tetracapsuloides bryosalmonae*, Tk—*Thelohanellus kitauei*, Mp—*Myxobolus pronini*, Mh—*Myxobolus honghuensis*, El—*Enteromyxum leei*, Ki—*Kudoa iwatai*, Sz—*Sphaeromyxa zaharoni*).

While we were able to find cytochrome C homologs in *Sphaeromyxa zaharoni*, *Enteromyxum leei*, and *Kudoa iwatai*, we found numerous stop codons and insertions within their predicted structural Cytochrome C domains. *Enteromyxum leei* and *Kudoa iwatai* have stop codons only within the above-mentioned insertions, whereas *Sphaeromyxa zaharoni* also has stop codons outside of them, within a highly conserved 69 aa region (Fig. 4).

Annotated mitochondrial genomes are available for myxosporeans *Enteromyxum leei*^35^ and *Kudoa iwatai*^36^. Despite the presence of stop-codons in their cytochrome C genes, genes of core subunits of complex IV and cytochrome b (related to complex III) are present in their mitochondrial genomes. They also possess the Pfam domains of respiratory subunits of complex III: PF00355 (Rieske) and PF02167 (Cytochrome C1). The latter statement is also true for *Sphaeromyxa zaharoni*, the third myxosporean with stop-codons in the cytochrome C gene. This suggests that the truncated cytochromes C of myxosporeans, resulting from these nonsense mutations, might still maintain their function in the respiratory chain.

### Not only Myxosporea, but also Malacosporea lack calpains, APAF-1, IAPs, and p53

**Figure 5:**
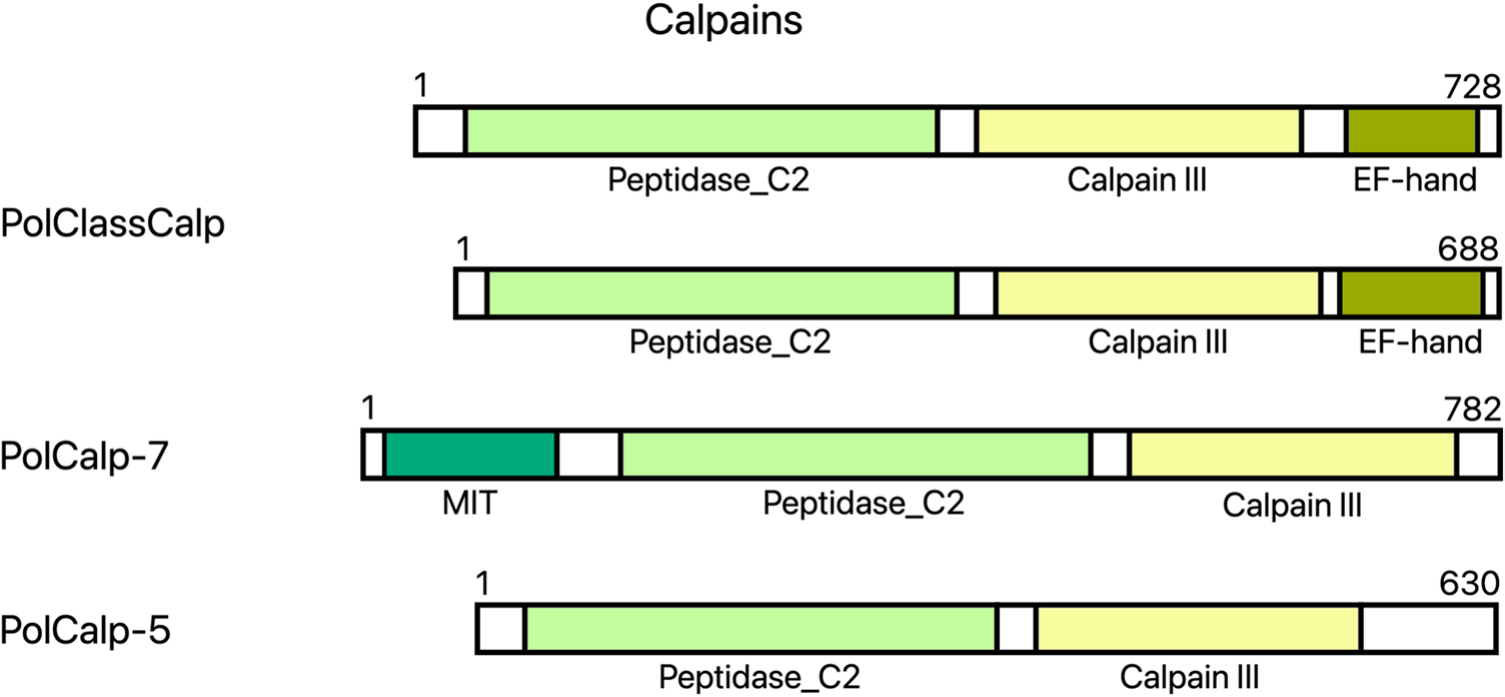
Predicted domain structures of calpains from *Polypodium* (Pol—*Polypodium hydriforme*, Calp—calpain, ClassCalp—classical calpain, Peptidase_C2—peptidase family C2 domain, MIT—microtubule interacting and transport domain). PolCalp-7 and PolCalp-5 share domain structure similarity with mammalian Calpain-7 and Calpain-5 respectively (the domain structure of mammalian calpains is shown in Luo et al.^40^).

Calpains are a family of proteases, some members of which are involved in caspase activation in response to calcium release from the endoplasmic reticulum^37^. The caspase activation is performed via the Peptidase_C2 and Calpain III peptidase domains, while the EF-hand domain is capable of binding calcium^38^. We were unable to find any calpain family members in Myxozoa, however, we found 4 putative calpains in the parasitic cnidarian *Polypodium* (Fig. 5). The domain structure of two of those calpains (presence of both peptidase domains and EF-hand domains) suggests their role in apoptosis. We refer to them as “classical” calpains as described in Croall and Ersfeld^39^, and in Luo et al.^40^. The EF-hand domain is important for calcium binding, whereas the Peptidase_C2 and Calpain III domains are homologous to peptidase domains capable of caspase activation.

**Figure 6:**
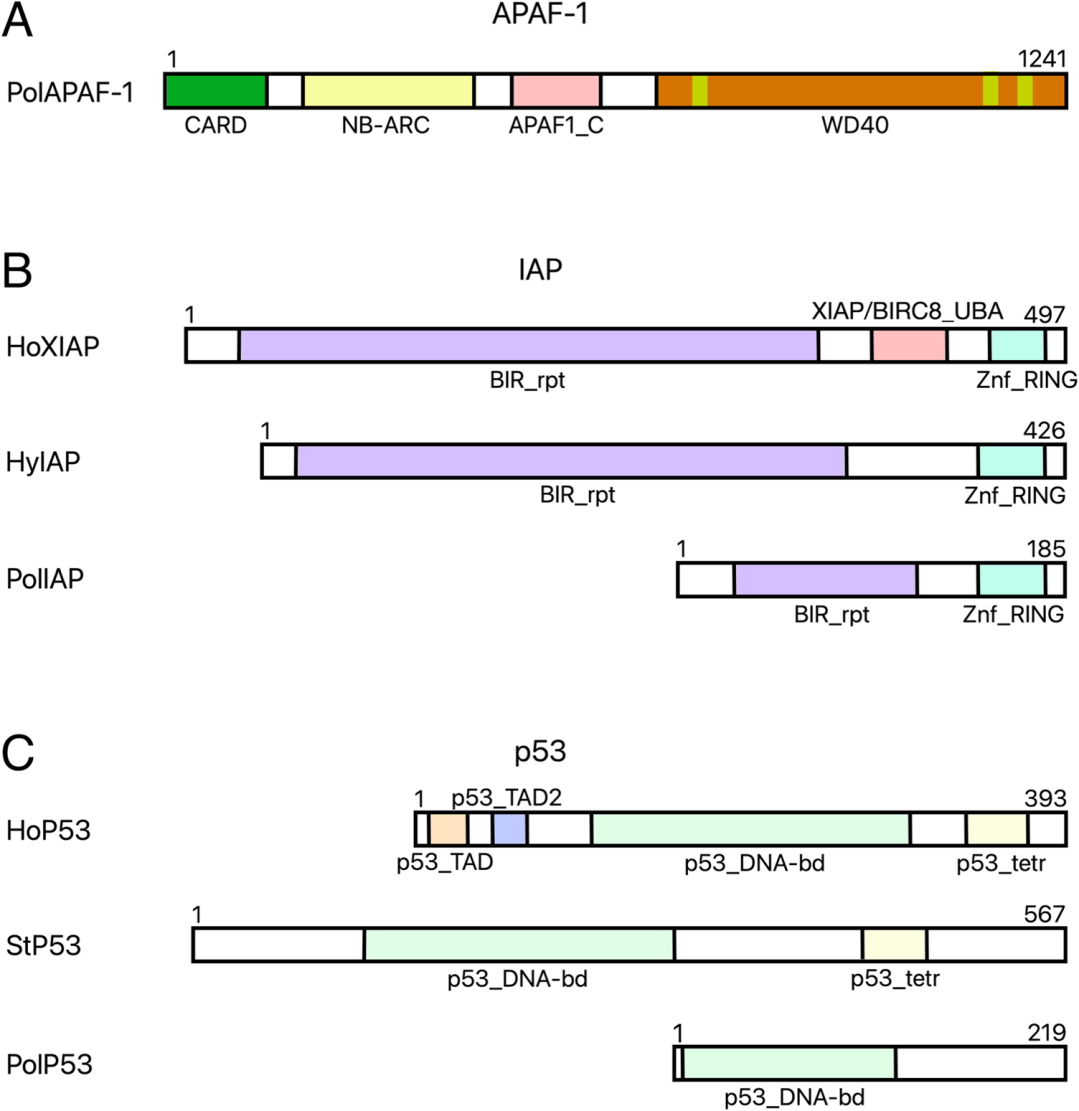
Predicted domain structures of APAF-1 from *Polypodium* (**A**), the predicted domain structure of partial IAP from *Polypodium* in comparison with homologs from *Hydra vulgaris* and *Homo sapiens* (**B**), the predicted domain structure of partial p53 from *Polypodium* in comparison with homologs from *Stylophora pistillata* and *Homo sapiens* (**C**) (Ho—*Homo sapiens*, Hy—*Hydra vulgaris*, St—*Stylophora pistillata*, Pol—*Polypodium hydriforme*, APAF-1—apoptotic protease-activating factor 1, IAP—inhibitor of apoptosis protein, NB-ARC—central nucleotide-binding domain of R proteins, APAF1_C—apoptotic protease-activating factor 1 C-terminal domain, BIR_rpt—Baculovirus inhibitor of apoptosis protein repeat, Znf_RING—RING-type zinc finger domain, p53_TAD—p53 transactivation domain, p53_TAD2—p53 transactivation domain 2, p53_DNA-bd—p53 DNA-binding domain, p53-tetr—p53 tetramerisation domain).

APAF-1 or apoptotic protease activating factor 1 is essential for forming the apoptosome when bound to cytochrome C, released from the mitochondria. Among studied parasitic Cnidaria only *Polypodium* has an APAF-1 homologue that we could identify. This homologue has the WD40 domain necessary for cytochrome C binding and the CARD domain for apoptosome formation (Fig. 6). This further suggests that *Polypodium* has functional apoptosis, while Myxosporea and Malacosporea do not.

IAPs are inhibitors of apoptosis. Among studied parasitic Cnidaria only *Polypodium* has an IAP. However, this predicted protein lacks the XIAP/BIRC8_UBA domain found in its human homolog and has a shorter BIR_rpt domain which binds Zn2+ and then binds the active site of caspases inhibiting their catalytic activity (Fig. 6). p53 is a transcription factor that is activated in response to a number of stressors including DNA damage and increases the expression of a number of pro-apoptotic genes (including APAF-1, Bak, Bax)^10^. The only parasitic Cnidaria in our analysis with p53 is *Polypodium*. However, the *Polypodium* p53 homolog is truncated and lacks some of the domains present in the human p53 (Fig. 6). It lacks the p53_TAD2 and p53_tetr domains which are involved in p53 transactivation and tetramerization respectively. The p53 DNA binding domain is present in *Polypodium*.

## Limitations

Our analysis relies on both gene presence and gene absence. The near-completeness of malacosporean transcriptomes is supported by the observation that almost all Pfam domains found in myxosporean genomes are also present in malacosporean transcriptomes (see Supplementary tables, Supplementary table S3).

Some genes can still be absent due to data incompleteness: when a gene is missing from the data, it might be due to insufficient read coverage. However, when this is the case, we expect different genes to be lost in independently sequenced related animals. For each species of Myxosporea we observe roughly the same set of lost apoptosis-related genes. A similar picture was observed for cancer-related gene loss of Myxosporean species as reported previously^13^.

## Conclusions

We evaluated the presence of core apoptotic proteins in free-living and parasitic Cnidaria. We observe a gradual loss of apoptosis-related proteins, starting with *Polypodium* who lost the major components of the extrinsic apoptotic pathway, followed by Myxozoa who lost the intrinsic apoptotic pathway as well. Compared to Myxosporea, Malacosporea retained one potentially functional caspase homolog (in *Buddenbrockia*). While both studied malacosporeans retained cytochrome C, the latter is missing in myxosporeans *Myxobolus squamalis*, *Henneguya salminicola* and its gene contains multiple stop-codons in *Kudoa iwatai*, *M. honghuensis*, and *Sphaeromyxa zaharoni*. However, the presence of other respiratory chain proteins suggests that the genes might be producing truncated, yet possibly functional cytochrome C proteins. Thus, Cnidaria present us with a unique opportunity to observe different stages of gradual loss of apoptosis. The observation that Malacosporea lost many genes involved in apoptosis regulation, weakens the previously proposed SCANDAL hypothesis that suggested a transmissible cancer origin for Myxosporea catastrophic simplification^13^.

Previously Panchin et al.^13^, did not observe an obvious trend for the loss of apoptosis and cancer-related genes in a subsample of parasitic Metazoa. A recent study of apoptosis in parasitic *Schistosoma*^41^ revealed the presence of most proteins typically involved in apoptosis. This makes the case of extreme loss of apoptosis-related proteins in Myxosporea and Malacosporea rather unique and not a necessary consequence of their parasitic life-style.

## Materials and methods

### Cnidaria genomes and transcriptomes

We used genomic data for *Myxobolus squamalis* GCA_010108815.1, *Henneguya salminicola* GCA_009887335.1, *Enteromyxum leei* GCA_001455295.2, *Sphaeromyxa zaharoni* GCA_001455285.1, *Kudoa iwatai* GCA_001407235.2, *Thelohanellus kitauei* GCA_000827895.1, *Myxobolus honghuensis* GWHBFXL00000000, *Hydra vulgaris* GCA_000004095.1, *Clytia hemisphaerica* GCA_902728285.1, *Nematostella vectensis* GCA_000209225, *Dendronephthya gigantea* GCA_004324835.1, *Acropora digitifera* GCF_000222465.1, and *Aurelia aurita* GCA_004194415.1. We additionally sequenced and assembled the genomes of *Polypodium hydriforme* and *Myxobolus pronini* (see Materials and methods, Sequence assembly). Assembly quality reports are provided in supplementary materials (Supplementary table S4). Transcriptomic data were available for *Tetracapsuloides bryosalmonae*, *Buddenbrockia plumatellae*, and *Polypodium hydriforme*. For *Tetracapsuloides bryosalmonae* we used a set of transcriptomic data, namely ready-made *T. bryosalmonae* assemblies from Kumar et al., 2021 (BioProject: PRJNA680464) and Faber et al.^22^ (BioProject: PRJEB19471), and *T. bryosalmonae* paired-end reads from Faber et al.^22^, Kumar et al.^23^, and Ahmad et al.^21^ (BioProject: PRJNA668017) which we assembled using SPAdes^42^ with k-mer lengths of 33, 49, and 93 (Table 2).

**Table 2:** Data sets which were used to make intersection transcriptomes.

Reads	Assembly
*T. bryosalmonae,* fish-derived^22^	*T. bryosalmonae,* bryozoan-derived^22^
*T. bryosalmonae,* fish-derived^22^	*T. bryosalmonae,* bryozoan-derived^23^
Kidney of *S. trutta* infected by *T. bryosalmonae*^21^	Genome of *S. trutta* (Genbank AC: GCA_901001165.2)

Reads were mapped on the ready-made assembly using Bwa mem algorithm and then mapped reads were assembled using SPAdes.

One of the approaches to filter contaminations of transcriptomic data of a parasitic animal is to compare its sequences from two hosts (as was done in Faber et al.^22^) or compare sequences from an infected and non-infected host of the same species. We created three filtered datasets using these approaches (Table 2). In two cases we used the intersection of RNA reads of *T. bryosalmonae* from a fish host and *T. bryosalmonae* assembly from a bryozoan host. In one case we used RNA reads from infected by *T. bryosalmonae* kidney of *Salmo trutta* and filtered out reads mapping to *S. trutta* reference genome. Reads were mapped on assemblies using Bwa mem algorithm^43^. After filtering, predicted *T. bryosalmonae* reads were assembled using SPAdes. SPAdes tool was used with k-mer lengths of 33, 49, and 93. All .sam and .bam files were processed with samtools package^44^.

### Exclusion of host contaminations in Myxozoan genomes and transcriptomes

In the eight available genomes of myxosporean species (*Myxobolus squamalis* GCA_010108815.1, *Henneguya salminicola* GCA_009887335.1, *Enteromyxum leei* GCA_001455295.2, *Sphaeromyxa zaharoni* GCA_001455285.1, *Kudoa iwatai* GCA_001407235.2, *Thelohanellus kitauei* GCA_000827895.1, *Myxobolus pronini* (see Materials and methods, Sequence assembly), *Myxobolus honghuensis* GWHBFXL00000000) we searched for possible host contaminations using an original Python script (see supplementary script genome_filter.py). Each contig from the genomes was used as a query for blastn-searches^26^ against a BLAST database built from the host genome. In the case of Myxosporea we used the publicly available genome of either the exact fish host or its closest available relative from Genbank (see Supplementary materials, List S1). If the best hit demonstrated > 85% identity and e-value < 1-e75, the query contig was excluded from the final assembly.

Another original python script (see supplementary script comparison.py) was used to remove host-derived contaminating sequences from the transcriptomes of malacosporeans *B. plumatellae* and *T. bryosalmonae*. First, we created two BLAST databases. The first consisted of several cnidarian transcriptomes, namely of *Aurelia aurita*, *Polypodium hydriforme*, *Hydra vulgaris*, *Pocillopora acuta*, *Myxobolus squamalis*, *Myxidium lieberkuehni*, and malacosporean transcriptomes that were obtained from hosts other than those which are being filtered. The second database consisted of a wide range of non-cnidarian organisms that may be regarded as additional probable contaminants. It was shown that possible contaminants include algae and cyanobacteria^23^, so we included *Tetraselmis striata* and *Nodularia spumigena* as representatives of these taxa. We also included the genome of bryozoan *Cristatella mucedo*, the transciptome of bryozoan *Fredericella sultana*, a transcriptome of another lophophorate—*Lingula anatina*, and also transcriptomes of *Drosophila melanogaster*, *Ruditapes philippinarium*, *Trichinella pseudospiralis*, *Salmo trutta*, *Danio rerio*, *Cricetulus griseus*. We found contaminations likely derived from these taxa when we manually checked BLAST search results of Malacosporean contigs. Such contaminations may sometimes occur when the same lab equipment is used for various sequencing projects or because bryozoans are filtrators and are susceptible to environmental contaminations.

Databases were created using translated ORFs obtained from the assemblies with the TransDecoder tool^45^, using a minimal ORF length of 70 amino acids. Each contig from malacosporean assemblies was searched against each of the two BLAST databases using blastp from the BLAST +^26^ tool package. Contigs were divided into three groups (“cnidaria”, “contamination” and “not clear”) based on e-value and bit score differences between the best hits against cnidarian and contaminant blast databases. If the e-value of the best cnidarian hit was at least 100 × less and its bit score at least 1.5 × more than that of the best hit from the contaminant dataset, this contig was considered as “cnidaria”. If the opposite was true, the contig was flagged as “contamination”. Otherwise, it was placed in the “not clear” group and subjected to further GC-content analysis (see below).

It was reported that Myxozoa have relatively lower GC-content compared to other Metazoa^46^. To visualize GC-content of each group of contigs we built a scatter plot with the length of contig and the GC-content on the Y- and X-axis respectively for “cnidaria”, “contamination”, and “not clear” groups of contigs. In the “cnidaria” group of contigs we obtained a set bounded by a bell curve with the average GC-content at 27–30% depending on the particular organism. The “contamination” group of contigs were placed as a flat bell-bounded set with average GC-content of 55%. In the “not clear” group we observed a distribution of GC-content indicating the presence of both “cnidaria” and “contamination” contigs (see Supplementary materials, Fig. S2) We used the following GC-content cutoffs: 39.03% for *B. plumatellae*, 40.80% for *T. bryoslamonae* assembly from fish kidney, 36.92% for *T. bryosalmonae* from bryozoan host to include the “not clear” contigs into the final “cnidaria” set of contigs.

Manual checks of potential contamination sources were conducted using BLAST searches and phylogenetic tree construction using IQ-TREE^47,48^ with further visualization in iTOL^49^.

### Identification of PFAM domains

We used the hmmscan tool from HMMER^27^ version 3.3.2 and the PFAM-A database (version 34.0)^50^ to identify PFAM domains in TransDecoder-predicted ORFs within the filtered contigs. This was done for all filtered Myxozoan genomes and transcriptomes, the *Polypodium hydriforme* genome, and several genomes of free-living Cnidaria genomes from the NCBI Genbank, namely *Acropora digitifera* GCF_000222465.1, *Aurelia aurita* GCA_004194415.1, *Clytia hemisphaerica* GCA_902728285.1, *Dendronephthya gigantea* GCA_004324835.1, *Hydra vulgaris* GCA_000004095.1, *Nematostella vectensis* GCA_000209225.

### Search for apoptosis-related domains and proteins

To elucidate the presence of core apoptotic proteins as functional multidomain units we conducted additional BLAST searches. Similar to Lasi et al.^7^, we adopted the list of proteins that are most important for apoptosis, namely caspase homologs, Bcl-2-family proteins, Apoptotic protease activating factor 1 (APAF-1), tumor necrosis factor receptor (TNF-R), inhibitor of apoptosis proteins (IAPs), Fas-associated protein with death domain (FADD), calpains, p53, and cytochrome C. For each protein we collected several reference cnidarian protein sequences from UniProt and NCBI Genbank, *Hydra* sequences from Lasi et al.^7^, were also included (see Supplementary materials, List S2, for list of accessions).

We performed blastp searches for each of the reference proteins against malacosporean (filtered and unfiltered *B. plumatellae*, *T. bryosalmonae* from invertebrate host, *T. bryosalmonae* from fish host with further manual analysis), *Polypodium hydriforme* genome and transcriptome, and also against filtered myxosporean genomes. Domain structures of identified hits were analyzed using the InterProScan tool^34^ with InterPro database^51^. Multiple alignments were built with the Muscle tool^52^ and visualized in the Jalview application^53^.

### Sample collection, DNA isolation

Specimens of *Carassius auratus gibelio* were caught in a pond near Barguzin River (53°69'N, 109°80'E) (Lake Baikal basin, Russia) in summer 2013. Round cysts of *Myxobolus pronini* were dissected from the body of fish and preserved in RNAlater for further analysis.

Total DNA was isolated from tissue samples by Diatom DNA Prep (IsoGene). One paired-end and two mate-pair libraries were prepared for *Myxobolus pronini* using the Nextera library preparation protocol (Illumina). The mate-pair libraries were prepared with the estimated mean insert lengths of 3500 bp and 7000 bp. The libraries were sequenced using an Illumina HiSeq 2000 instrument, generating 163 M 100 bp read pairs for the paired-end library, 31 M read pairs for the 3500 bp mate-pair library and 38 M read pairs for the 7000 bp mate-pair library.

Specimens of *Acipenser gueldenstaedtii* were caught by A.A. Gerasimov in the Volga River delta near the fall of the river into the Caspian Sea (region of city of Astrakhan) (46°16′N, 47°57′E) in 2013. This was done by A.A. Gerasimov as part of an official fish conservation practice. This practice involves catching a small number of fish, extraction of their oocytes for further breeding, followed by the release of fry back into their wild habitat. Stolons of *Polypodium hydriforme* were surgically isolated from infected oocytes of *Acipenser gueldenstaedtii* host, washed, and preserved in RNAlater for further analysis.

Total DNA was isolated from stolon samples by Diatom DNA Prep (IsoGene). One paired-end library was prepared for using the TruSeq library preparation protocol (Illumina). The *Polypodium* library was sequenced using an Illumina HiSeq 2500 instrument, generating 260 M read pairs with the read length of 250 bp.

### Sequence assembly

The sequencing reads for *Myxobolus pronini* and *Polypodium hydriforme* were trimmed using Trimmomatic^54^ to remove the adapters. Prior to assembly, the *Myxobolus pronini* reads were processed using the error correction utility of the ALLPATHS-LG program^55^. The assembly for *Myxobolus pronini* data was performed using Velvet^56^ with a k-mer size of 55. Scaffolds obtained with Velvet were then processed using the Redundans pipeline^57^ with an identity threshold of 0.85 to obtain the final assembly draft. The assembly of *Polypodium* data was performed using SPAdes^42^ with three k-mer sizes: 77, 99, 127. The assemblies were evaluated using QUAST^58^.

## Supplementary Information

Supplementary Information 1.

Supplementary Information 2.

Supplementary Information 3.

Supplementary Information 4.

Supplementary Information 5.

## Data Availability

The datasets supporting the conclusions of this article are included within the article (and its additional files). The sequenced genomic data are available under the following accession numbers: *Myxobolus pronini*: NCBI BioProject: PRJNA957449, NCBI SRA: SRR24216800, SRR24216801, SRR24216802, SRR24216803, SRR24216804; *Polypodium hydriforme*: NCBI BioProject: PRJNA957841, NCBI SRA: SRR24234439. The sequences derived from sequencing data are available in the NCBI GenBank repository under the following accession numbers: WEC89223, WEC89224, WEC89225. The fasta-file with all sequences predicted and described in this article (including sequences derived from publicly available sequencing data) is accessible in the Figshare repository under the following link: https://figshare.com/articles/dataset/Apoptotic_gene_loss_in_Cnidaria_is_associated_with_transition_to_parasitism/21719930.
