# Supplementary material for: Apoptotic gene loss in Cnidaria is associated with transition to parasitism
Source: Sci Rep. 2023 May 17;13:8015. doi: 10.1038/s41598-023-34248-y (PMC10192318; doi:10.1038/s41598-023-34248-y)
Supplement: Supplementary file 3 — Supplementary Information 3. [file 41598_2023_34248_MOESM3_ESM.docx]

**Supplementary files legend**

All predicted apoptosis-related proteins of parasitic Cnidaria are stored in the file “Proteins.fa” in fasta-format.

Both python-scripts utilized in this research are stored in the folder “Scripts”. Script “genome_filter.py” was utilized for filtration of contaminations in myxosporeans’ genomes, script “comparison.py” was utilized for filtration of contaminations in malacosporeans’ transcriptomes.

Supplementary figures and lists are stored in the file “Supplementary_materials.docx”. Figure S1 shows a phylogenetic tree of Bcl-2 family proteins, figure S2 shows an example of transcriptome filtration based on GC-content (example for *Buddenbrockia plumatellae*). Figure S3 shows a phylogenetic tree for caspases of parasitic Cnidaria and *Hydra*. List S1 consists of accession numbers of fish species’ genomes used for filtration of fish-derived contaminations in myxosporeans’ genomes. List S2 consists of reference proteins used in comparative BLAST analysis to predict their homologs in parasitic Cnidaria.

All supplementary tables are stored in the file “Supplementary_tables.xlsx”. Numbers of hits of Pfam domains considered in SCANDALs article [11] in filtered genomes of Myxosporea, filtered transcriptomes of Malacosporea and genomes of the other Cnidaria are presented in the Supplementary table S1. Number of hits of Pfam domains related to apoptosis according to Pfam keyword search in filtered genomes of Myxosporea, filtered transcriptomes of Malacosporea and genomes of the other Cnidaria are presented in the Supplementary table S2. Comparison of sets of domains predicted in Myxosporea and Malacosporea is shown in the Supplementary table S3. All predicted Pfam domains were considered. In the first two columns (yellow) there is a number of Pfam domains presented in each group of Myxozoa. Two avoid possible aberrations, we took into consideration domains which are presented in at least half of members of respective group (in at least 4 out of 8 myxosporeans and in at least 1 out of 2 malacosporeans). In three other columns (blue) there is a size of all possible intersections between these two sets of domains. Note that the number of domains which are presented in Myxosporea, but not in Malacosporea many times less than number of domains which are presented in Malacosporea, but not in Myxosporea. In Supplementary table S4 we provide a quality report on genomes of parasitic cnidarians that we used in this study.
