# Supplementary material for: Apoptotic gene loss in Cnidaria is associated with transition to parasitism
Source: Sci Rep. 2023 May 17;13:8015. doi: 10.1038/s41598-023-34248-y (PMC10192318; doi:10.1038/s41598-023-34248-y)
Supplement: Supplementary file 4 — Supplementary Information 4. [file 41598_2023_34248_MOESM4_ESM.docx]

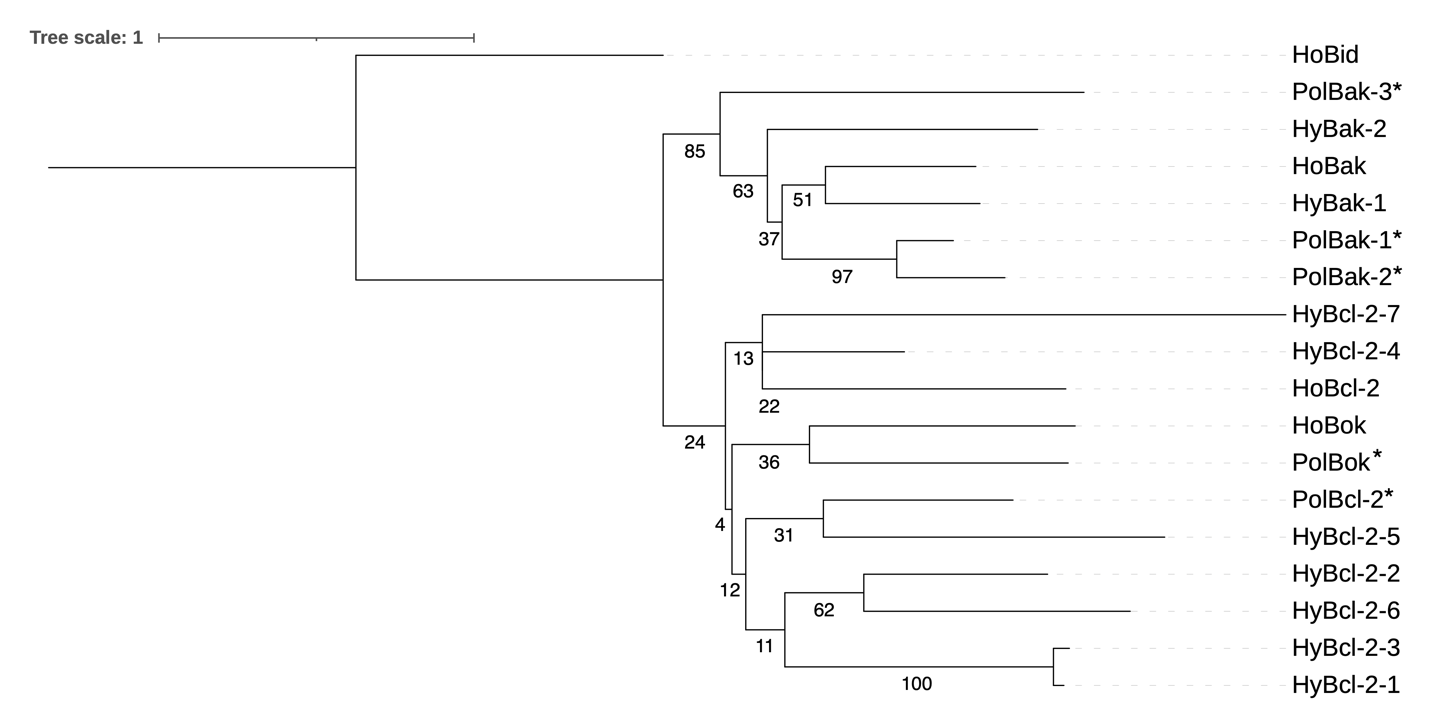


**Figure S1.** Phylogenetic tree of BCL-2 family proteins from *Polypodium hydriforme*, *Hydra vulgaris*, and *Homo sapiens*, which suggests the possible role of proteins from *P. hydriforme*. Considered proteins are marked with *. According to this tree we suggest that *P. hydriforme* have three homologs of Bak, one homolog of Bcl-2, and one homolog of human’s Bok. The tree was constructed using IQTree tool^47,48^ with ModelFinder (chosen model is LG+G4). Numbers on nodes represent a standard non-parametric bootstrap (Bootstrap + ML tree + consensus tree) with 100 replicates. (Pol – *Polypodium hydriforme*, Hy – *Hydra vulgaris*, Ho – *Homo sapiens*)


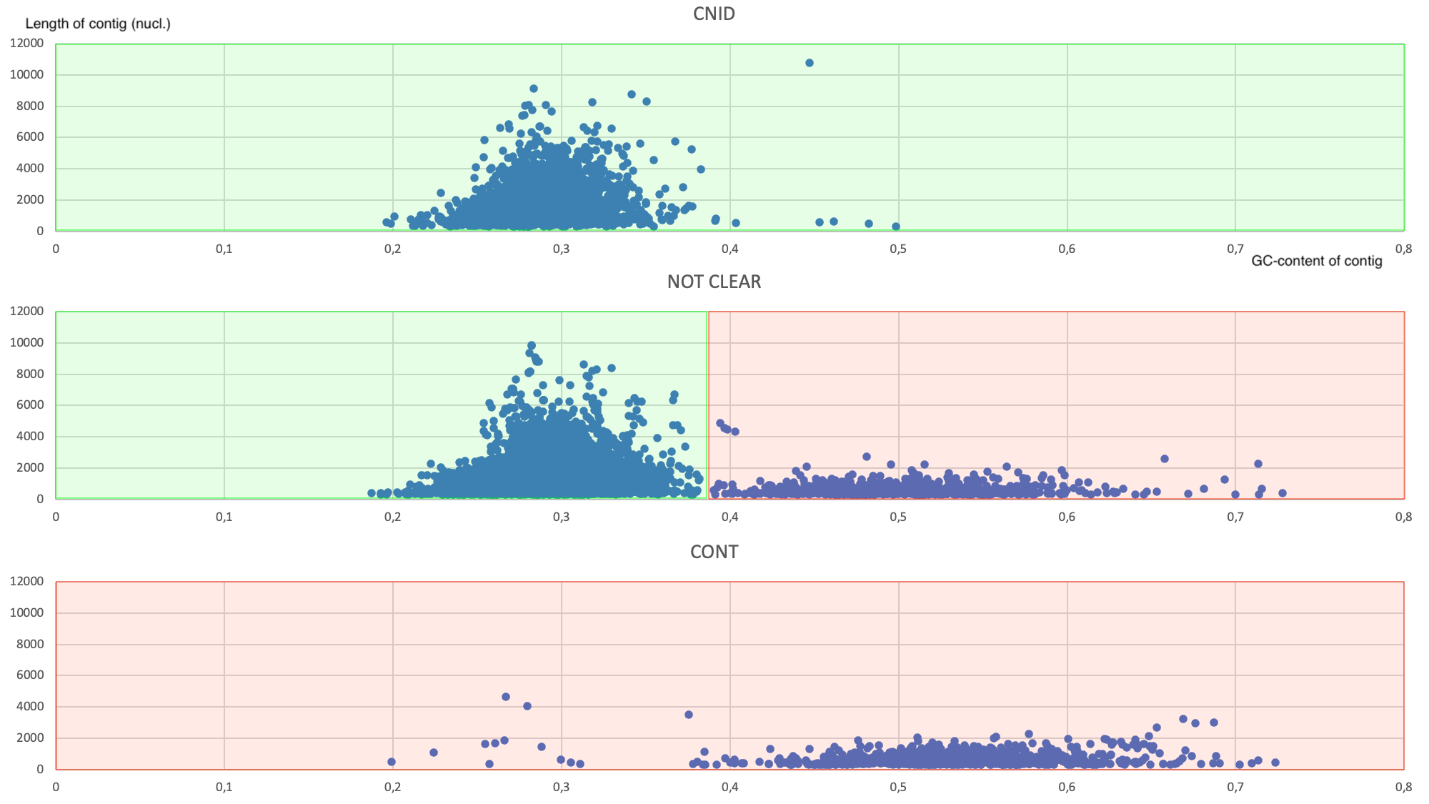


**Figure S2**. *Buddenbrockia plumatellae* contigs’ length distribution in dependence of GC-content of respective contig. Contigs are divided into three groups after BLAST analysis (“CNID” – most likely belong to Cnidaria, “CONT” – most probable contaminations, “NOT CLEAR” – do not clearly belong to above mentioned groups). In “NOT CLEAR” group suggested contigs of cnidarian parasite are distinguished according to low GC-content and separated by the visible gap. The green color shows contigs which will be further considered as cnidarian, the red color shows suggested contaminations.


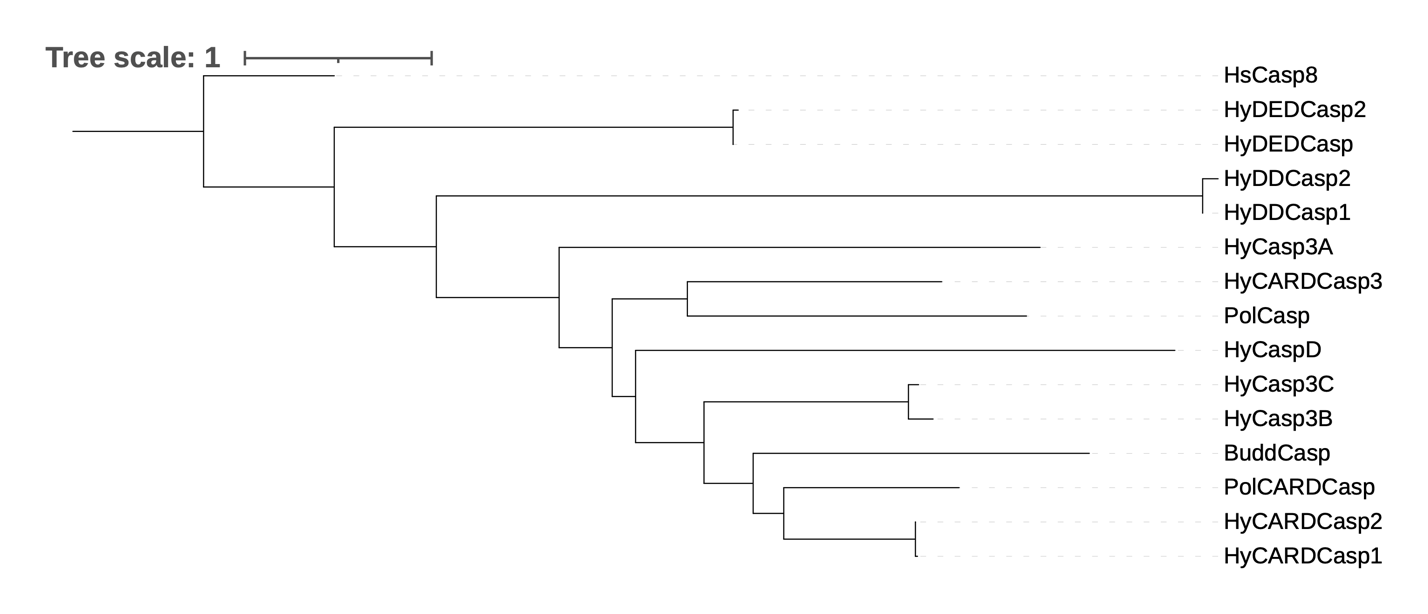


**Figure S3.** Phylogenetic tree of caspases from *H. vulgaris*, *P. hydriforme*, and *B. plumatellae*. The tree was constructed using the IQTree tool^47,48^ with ModelFinder (chosen model is WAG+F+R3).

**List S1.** Genomes of host species or the closest available relative, which were used to filter contaminations in genomes of Myxosporea.

GCA_015220745.1 *Sebastes umbrosus*

GCA_900880675.1 *Sparus aurata*

GCA_018340385.1 *Cyprinus carpio*

GCA_013265735.3 *Oncorhynchus mykiss*

GCA_002021735.2 *Oncorhynchus kisutch*

**List S2.** Accession numbers in NCBI Genbank and UniProt databases of key apoptosis proteins, which were used in BLAST analysis to identify their homologs in Myxozoa and *Polypodium*.

BCL2-family:

EF104645.1_1

EU035760.1_1

EF104646.1_1

EF104647.1_1

EU035765.1_1

EU035764.1_1

EU035763.1_1

EU035762.1_1

EU035761.1_1

A0A6S7I151_PARCT

A0A2B4RIL0_STYPI

A0A6P8IEP5_ACTTE

A7LM80_HYDVU

A1E3K7_HYDVU

A0A2B4RCQ3_STYPI

A0A6P8HSD1_ACTTE

T2MD83_HYDVU

A0A6P8H8E3_ACTTE

A0A6P8HH69_ACTTE

A0A6S7HWP1_PARCT

T2MBP4_HYDVU

A7LM79_HYDVU

A0A2B4SGV3_STYPI

A7LM78_HYDVU

A0A6S7G6P7_PARCT

A0A6S7KD98_PARCT

A0A2B4SG37_STYPI

A0A6P8I8Z0_ACTTE

A1E3K6_HYDVU

A7LM81_HYDVU

A0A7D7PV32_FIMAN

A0A7D9I3R2_PARCT

BBC3_HUMAN

BID_MOUSE

B2L11_HUMAN

BMF_HUMAN

BIK_HUMAN

BIK_MOUSE

BNIP3_HUMAN

A0A2B4SMJ0_STYPI

Caspases:

A0A6P8HM59_ACTTE

A0A6P8HZ29_ACTTE

E2DGP9_HYDVU

A0A2B4RUQ2_STYPI

A0A2B4RVA9_STYPI

A0A2B4SCW1_STYPI

A0A2B4RVR5_STYPI

A0A6P8IR53_ACTTE

A0A6P8IT18_ACTTE

A0A2B4SMB4_STYPI

A0A2B4SLD3_STYPI

F2XX04_HYDVU

D1MAR5_HYDVU

A0A2B4SWS7_STYPI

A0A0B5KPL7_ACRMI

A0A0B5KG64_ACRMI

D1MAR3_HYDVU

Q09Y99_ANEVI

A0A7D9DFR5_PARCT

A0A7D9HBI3_PARCT

Q3HL92_EXADI

A0A6P8IQR9_ACTTE

A0A6P8J0G4_ACTTE

A0A6P8IJ59_ACTTE

A0A2B4SF88_STYPI

A0A7D9DWX4_PARCT

A0A7D7PPD2_FIMAN

A0A7D7KP96_FIMAN

A0A7D9MA73_PARCT

D1MAR2_HYDVU

A0A6P8H7R6_ACTTE

A0A6P8I1H3_ACTTE

A0A6P8IUV3_ACTTE

A0A6P8HSQ5_ACTTE

A0A6P8IUC4_ACTTE

A0A6P8I241_ACTTE

A0A6P8IAT5_ACTTE

A0A6P8I0R6_ACTTE

A0A6P8HZT4_ACTTE

A0A6P8IKX8_ACTTE

A0A6P8HTB3_ACTTE

A0A7D7PS49_FIMAN

A0A7D9M8D8_PARCT

A0A7D9K1Z6_PARCT

B1GXL8_HYDEC

A0A7D9LTJ6_PARCT

A0A2B4SVG3_STYPI

A0A288MWZ7_POCDA

A0A2B4S6K1_STYPI

A0A2B4S7Z0_STYPI

A0A2B4SRJ7_STYPI

A0A2B4SJP8_STYPI

A0A2B4SL76_STYPI

G8XQY3_STYPI

A0A2B4SK08_STYPI

A0A2B4RBN1_STYPI

A0A2B4S5A8_STYPI

A0A2B4R674_STYPI

A0A2B4ST76_STYPI

A0A2B4SPF7_STYPI

A0A2B4RVA3_STYPI

A0A2B4R267_STYPI

A0A0X9PYG6_ACRMI

D2KFF8_HYDVU

Q9GV88_HYDVU

Q9GV89_HYDVU

Q5D0W5_HYDVU

D1MAR4_HYDVU

F2XX05_HYDVU

A0A7D9LC40_PARCT

A0A7D9M843_PARCT

A0A6S7HWU7_PARCT

A0A6S7GY62_PARCT

A0A7D9LT33_PARCT

A0A7D9LAK1_PARCT

A0A7D9D6J0_PARCT

A0A6S7JMD7_PARCT

A0A6S7FTR5_PARCT

A0A7D9DN84_PARCT

A0A7D9DID2_PARCT

A0A6S7KA37_PARCT

A0A6S7KPK6_PARCT

A0A7D9MCZ9_PARCT

A0A6S7HE82_PARCT

A0A7D9HX38_PARCT

A0A6S7JXR5_PARCT

A0A6S7HEX1_PARCT

A0A7D9HN77_PARCT

A0A7D9HYG7_PARCT

A0A6S7KDD2_PARCT

A0A7D9EV71_PARCT

A0A6S7JYZ6_PARCT

A0A7D9L0N6_PARCT

A0A6S7IMR9_PARCT

A0A6S7JXR0_PARCT

A0A7D9LMA6_PARCT

A0A7D9L4P0_PARCT

A0A6S7KEB8_PARCT

D7PPM4_ACRPL

XP_001633895.1

XP_032226627.1

XP_001623955.2

XP_032238419.1

XP_032239949.1

XP_001631692.2

XP_001626560.2

XP_032230667.1

AF155128.1_1

AF155127.1_1

AY924233.1_1

GU128636.1_1

GU128637.1_1

GU128638.1_1

GU128639.1_1

GU208869.1_1

Calpains:

T2MJ45_HYDVU

A0A2B4R5D8_STYPI

A0A2B4RSD3_STYPI

A0A2B4RV23_STYPI

A0A6P8H968_ACTTE

T2M2K0_HYDVU

T2MI17_HYDVU

T2MIT4_HYDVU

APAF-1:

GU121227

A0A0U1YWJ4_ACRMI

Cytochrome C:

A0A2B4SE09_STYPI

A0A6P8IQD5_ACTTE

J7QAM2_EXADI

A0A6S7J8C7_PARCT

and our predicted genes from other myxosporeans and malacosporeans

Fas:

A0A2B4SGJ8_STYPI

A0A2B4RTW5_STYPI

A0A0B5KML6_ACRMI

A0A7D9EH90_PARCT

A0A8B7DK60_HYDVU

A0A8B7DIG3_HYDVU

FADD:

A0A0B5KML6_ACRMI

A0A6P8IU21_ACTTE

XP_002166467.1

IAPs:

XIAP_HUMAN

XIAP_RAT

XIAP_HUMAN

XIAP_RAT

A0A2B4SSM4_STYPI

A0A6P8IDW6_ACTTE

A0A8B7DXA5_HYDVU

T2MEB3_HYDVU

BIRC3_MOUSE

BIRC2_MOUSE

GU121225.1_1

p53:

A0A2B4RUK1_STYPI

A0A2B4SCF0_STYPI

P53_MOUSE

P53_HUMAN

CEP1_CAEEL
